# Deciphering meropenem persistence in *Acinetobacter baumannii* facilitates discovery of anti-persister activity of thymol

**DOI:** 10.1128/aac.01381-24

**Published:** 2025-02-20

**Authors:** Arsalan Hussain, Timsy Bhando, Ananth Casius, Rinki Gupta, Ranjana Pathania

**Affiliations:** 1 Department of Biosciences and Bioengineering, Indian Institute of Technology Roorkee 30112 , Roorkee, India; Columbia University Irving Medical Center, New York, New York, USA

**Keywords:** antibiotic resistance, anti-persister, thymol, spontaneous persisters, multi-drug tolerance

## Abstract

Decades of antibiotic misuse have accelerated the emergence of multi- and extensively drug-resistant bacteria. Bacterial pathogens employ several strategies such as antibiotic resistance, tolerance, and biofilm formation in response to extreme environments and antibiotic stress. Another crucial survival mechanism involves the stochastic generation of bacterial subpopulations known as persisters, which can endure high concentrations of antibiotics. Upon removal of antibiotic stress, these subpopulations revert back to their original phenotype which links them to the relapse and recalcitrance of chronic infections, a significant problem in clinical settings. Persistent infections are particularly notable in *Acinetobacter baumannii*, a top-priority ESKAPE pathogen, where carbapenems serve as last-resort antibiotics. Several reports indicate the rising therapeutic failure of carbapenems due to persistence, underscoring the importance of developing anti-persister therapeutics. In this study, we explored the mechanisms of transient persister formation in *A. baumannii* against meropenem. Our investigation revealed significant changes in membrane properties and energetics in meropenem persisters of *A. baumannii*, including a noteworthy increase in tolerance to other antibiotics. This understanding guided the evaluation of an in-house collection of GRAS status compounds for their potential anti-persister activity. The compound thymol demonstrated remarkable inhibitory activity against meropenem persisters of *A. baumannii* and other ESKAPE pathogens. Further investigation revealed its impact on persister cell physiology, including efflux pump inhibition and disruption of cellular respiration. Given our results, we propose a compelling strategy where thymol could be employed either as a monotherapy or in combination with meropenem in anti-persister therapeutics.

## INTRODUCTION

The overconsumption and abuse of antibiotics have triggered an emergence of multidrug resistant (MDR) and extensively drug-resistant (XDR) bacterial pathogens. This has led to an estimated 4.95 million deaths per year globally according to the World Health Organisation (WHO) (1, 2). Gram-negative bacteria including *Acinetobacter baumannii, Klebsiella pneumoniae, Pseudomonas aeruginosa,* and *Enterobacter* spp. categorized among the ESKAPE group that pose significant threat in clinical settings (3). A spectrum of antibiotic-resistant mechanisms and intricate membrane structure make them challenging to treat with the existing antibiotic arsenal (4). Given the substantial incorporation of resistance conferring genes in the repository of resistance determinants, alternative strategies are urgently needed to address this critical challenge (5).

Adding to the distress, the escalating global crisis of antibiotic resistance is aggravated by the presence of phenotypic heterogeneity within bacterial populations (6). This allows them to evade conventional antibiotic treatment leading to relapse and recurrence of infections in clinical settings. Among the gram-negative ESKAPE pathogens, *A. baumannii* is responsible for causing wide range of recurring skin and soft tissue infections, meningitis, ventilator-associated pneumonia (VAP), and chronic urinary tract infections both in the community and hospital settings (7). Besides the wide array of infection, and notably high level of intrinsic resistance, *A. baumannii* displays remarkable genetic adaptability to diverse environmental stress conditions (8). Moreover, this pathogen demonstrates exceptional ability for nosocomial transmission, coupled with resistance to desiccation and propensity to form biofilm on medical devices and surfaces (9). Additionally, *A. baumannii* can enter a state of reversible transient dormancy, also known as spontaneous persisters, allowing it to withstand high antibiotic concentrations (10). This specific phenomenon of spontaneous persistence occurs during active growth phases in response to environmental stress such as antibiotic exposure which leads to temporary quiescence (11). As a result, this phenomenon enables them to survive antibiotic treatment and later return to a normal state, often causing recurrence of infection (6).

Carbapenems, including meropenem, are currently the safest last resort treatment option for infections caused by *A. baumannii* (12). However, the increasing prevalence of both resistant isolates and persister phenotypes have limited their effectiveness (13). Understanding the mechanisms behind persister formation will facilitate the discovery of novel therapeutic agents that inhibit persister populations, providing a successful alternative to treat persistent *A. baumannii* infections (14). The development of anti-persister therapy can encompass strategies that enables direct eradication of existing persister population or prevention of persister cell formation (15). The dormant phenotype and diminished metabolic activity contribute to temporary inactivation of the macromolecular targets of antibiotics in persister populations (16). As a result, these populations rely heavily on an intact membrane to maintain cellular viability (17). Therefore, targeting components of the bacterial membrane and proteins essential in membrane functions and energetics are currently considered ideal targets for anti-persister therapy (14). Recent studies have shown the emergence of membrane active agents as new means to eradicate bacterial persisters by generation of hydroxyl radicals (18), targeting membrane functions and antibiotic efflux pumps (19).

In this study, we investigated the characteristics underlying persister formation upon meropenem treatment during the exponential growth phase of *A. baumannii*. In light of these findings, we also observed that meropenem persisters of exhibited tolerance to clinically relevant antibiotics belonging to different classes, including rifampicin, tigecycline, and levofloxacin. Consequently, we performed a mechanism-based identification of compounds with potent anti-persister activity. A panel of Generally Regarded as Safe (GRAS) status compounds was assessed for their potential to target bacterial membrane, increase reactive oxygen species (ROS) production, and inhibit the proton motive force (PMF) and multidrug efflux pumps. Thymol was identified to exhibit excellent inhibitory activity against *A. baumannii* persisters upon meropenem treatment and other clinically relevant classes of antibiotics. Furthermore, exploiting the persister-suppressing ability of thymol treatment demonstrated promising efficacy in an *in vivo* murine wound infection model of meropenem persister of *A. baumannii*. To our knowledge, this study is among the first to employ a mechanism-based identification of novel anti-persister compounds against *A. baumannii*. Notably, we report the discovery of GRAS status natural compounds that show promising inhibitory activity against *A. baumannii* persisters. These findings hold significant implications for enhancing the treatment of recalcitrant *A. baumannii* infections in clinical settings.

## RESULTS

### Meropenem treatment elicits high persister cell formation frequency in *Acinetobacter baumannii* AYE

**Fig 1:**
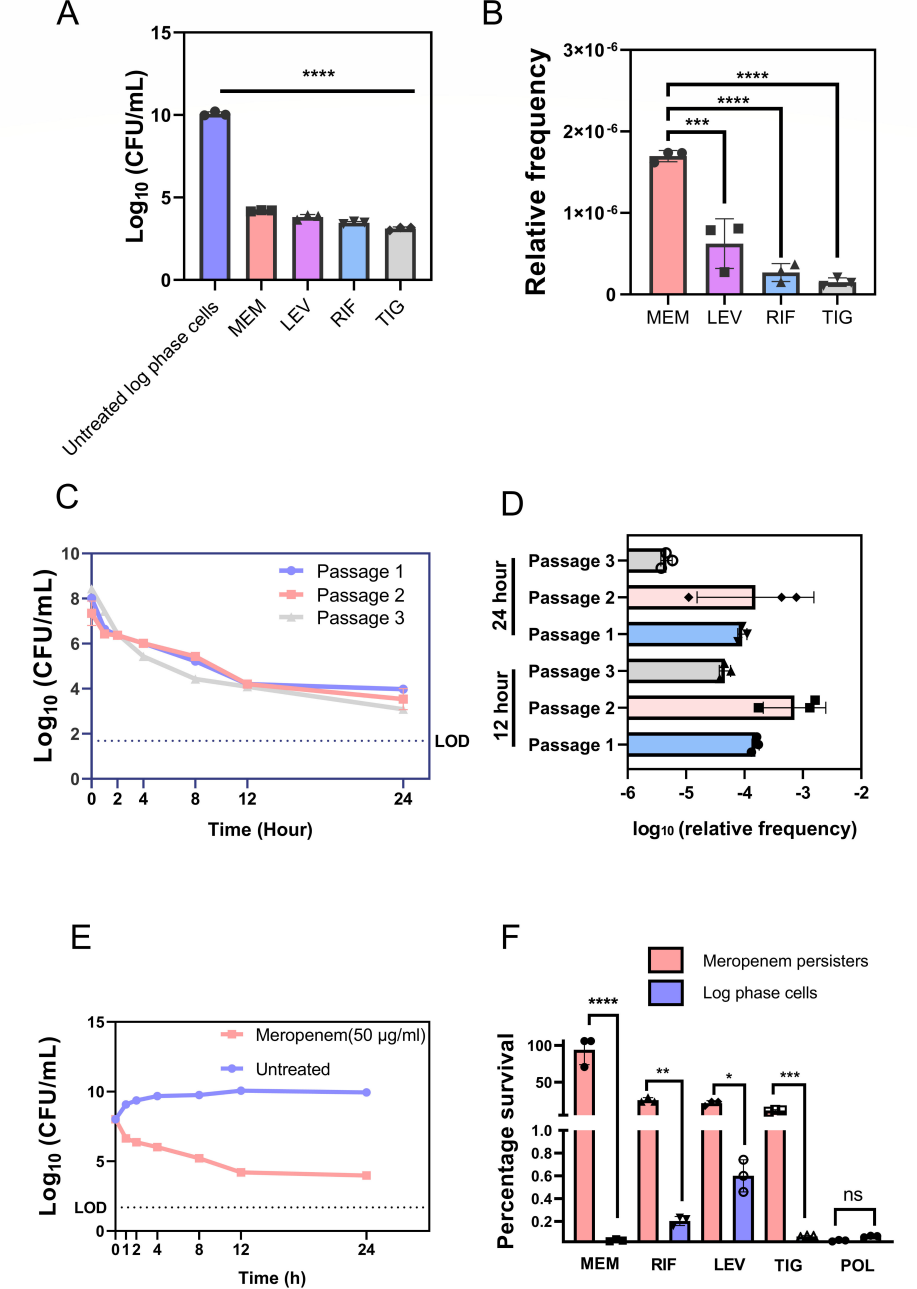
(**A**) *A. baumannii* AYE formed persister in response to meropenem (MEM, 50 µg/mL), tigecycline (TIG, 12.5 µg/mL), rifampicin (RIF, 300 µg/mL), and levofloxacin (LEV, 150 µg/mL), 12 h post treatment. (**B**) Relative frequency of persister formation to different antibiotics as compared to log phase cells; calculated as follows: number of colonies obtained after treatment/number of colonies obtained in untreated log phase cells. (**C**) Non-heritability of the persister cells formed against meropenem. Overnight culture of *A. baumannii* AYE was inoculated in LB broth to obtain log phase cells and exposed to meropenem (50 µg/mL) for 24 h. The surviving persister cells were regrown in antibiotic-free LB broth for 24 h and re-exposed to meropenem (50 µg/mL) for 24 h. The procedure was repeated for three consecutive passages (Passage 1, Passage 2, and Passage 3). (**D**) Frequency of persister cell formation following each passage was determined to check the level of persister formation. (**E**) Meropenem persisters of *A. baumannii* AYE reveal biphasic killing pattern. (**F**) Percentage surviving fraction of meropenem persisters of *A. baumannii* AYE in the presence of meropenem (50 µg/mL), rifampicin (40 µg/mL), levofloxacin (40 µg/mL), tigecycline (10 µg/mL), and polymyxin B (2.5 µg/mL) at 8 h post treatment. LOD—limit of detection. Each value represents the mean of three values and error bars indicate standard error. Significance determined by one-way ANOVA followed by Tukey’s multiple comparison test (*P < 0.01; **P < 0.001; ***P < 0.003; ****P <0.0001; ns, non-significant).

The minimum inhibitory concentration of different antibiotic classes was determined against multi-drug resistant isolate *A. baumannii* AYE, as well as other strains (refer to Table S1 for the list of strains and Table S2 for MIC values). We assessed persister cell formation in *A. baumannii* AYE in the presence of four bactericidal antibiotics including meropenem, tigecycline, rifampicin, and levofloxacin using concentrations determined as the minimum required to induce persister formation, as detailed in the supplemental material and depicted in Fig. S1. We observed significant fraction of surviving persisters after 12 h of treatment individually with all four antibiotics, which is represented by the number of surviving colonies (Fig. 1A). From these data, we derived the frequency of persister cell formation which was quantified relative to untreated log phase cells (Fig. 1B). Among the antibiotics tested, meropenem exhibited the highest relative frequency of persister formation −1.69 × 10^−6^, followed sequentially by levofloxacin (6.23 × 10^−7^), rifampicin (2.68 × 10^−7^), and tigecycline (1.52 × 10^−7^) (Fig. 1B). Meropenem, which is a beta-lactam antibiotic disrupts cell wall synthesis thereby inducing an immediate structural instability. Based on these findings, we observed that exposure to meropenem significantly increases the formation of a persister cell population. Given the critical role of meropenem as a last-resort antibiotic, these findings underscore the importance of selecting meropenem for further investigation in our study. We initially assessed the heritability of meropenem persisters (20). Isolated persisters post-treatment were cultured in fresh medium and passaged for up to three generations (21). Cultures derived from persister cells were as sensitive to meropenem as the parent culture and displayed similar killing patterns upon treatment (Fig. 1C), confirming the non-heritability of persistence (21). Additionally, the relative frequency of persister cell formation after every 12 and 24 h were determined for each passage. It was observed that the frequency remained consistent across all passages (Fig. 1D). This suggests that persister formation is a transient state rather than a genetically inherited trait (22).

### Meropenem persisters of *A. baumannii* AYE demonstrate biphasic killing pattern and exhibit multi-drug tolerance

Bacterial persistence is characterized by a biphasic killing pattern under bactericidal antibiotic concentrations (23). Initially, there is a dramatic reduction in the bulk of the bacterial population, followed by a plateau phase representing the surviving persister population (24). This biphasic killing pattern is observed to be both time and concentration-dependent (23). We subjected log-phase cells of *A. baumannii* AYE to bactericidal concentrations of meropenem (50 µg/mL; 100× MIC). This resulted in a rapid reduction in viable cell counts, with a decrease of 3.82 ± 0.01 log units in CFU within the first 12 h (Fig. 1E). Following this initial decline, the CFU remained relatively constant up to 24 h (Fig. 1E). This biphasic killing pattern indicates the presence of persister cells upon treatment with meropenem (25). These cells exhibit transient dormancy which allows them to survive antibiotic stress (26) and potentially revert to a susceptible state once the stressor is removed (27).

Since persisters exhibit a transient state of dormancy, they are known to show tolerance to multiple antibiotics due to a possible reduction in drug target activity (28). Consequently, we sought to investigate the cross-tolerance of meropenem persisters of *A. baumannii* AYE, toward antibiotics targeting various cellular pathways (29). Cross-tolerance was assessed by exposing isolated meropenem persisters to rifampicin, tigecycline, levofloxacin, and polymyxin B, for 12 ho (29). The surviving fraction of meropenem persisters exposed to these antibiotics was compared to the survival fraction of log phase cells. The results indicate that meropenem persister population exhibits significant tolerance to these antibiotics compared to log-phase cells (Fig. 1F). Interestingly, we did not observe cross-tolerance to polymyxin B in meropenem persisters, which highlights its potential viability as a treatment option. This lack of cross-tolerance may be attributed to the metabolism-independent activity and membrane destabilization effects, which are characteristic of polymyxin-class antibiotics (30). However, given the status of polymyxin B as a last-resort antibiotic, along with the documented persister phenomenon in *A. baumannii* and its associated nephrotoxicity, its effectiveness as an anti-persister agent is limited (31). Hence, we aimed to elucidate the physiological characteristics of meropenem persisters in order to design a strategy for identifying potent anti-persister compounds that may target multiple survival mechanisms.

### Perturbation of the proton motive force leads to depletion of intracellular ATP levels in meropenem persisters

**Fig 2:**
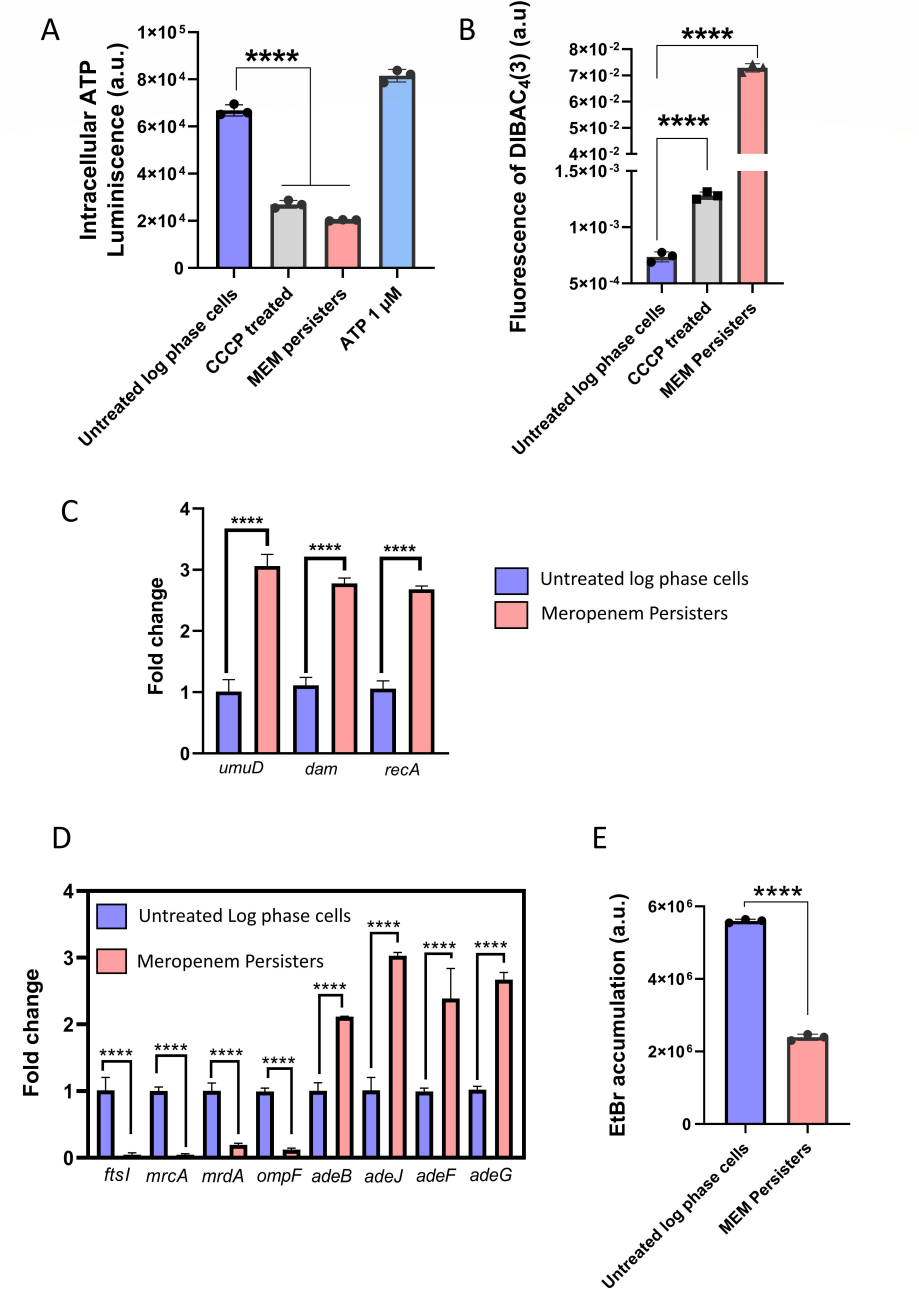
(**A**) Measurement of intracellular ATP levels in untreated log phase cells and meropenem persisters of *A. baumannii* AYE. (**B**) Measurement of the membrane potential component Δ*ψ*, using the membrane potential-sensitive fluorescent probe DiBAC_4_(3) in untreated log phase cells and meropenem persisters of *A. baumannii* AYE. (**C**) qRT-PCR analysis of SOS-response genes in *A. baumannii* AYE. Relative gene expression was calculated by the ΔΔ*C_T_* method, using 16S rRNA as the reference genes. (**D**) qRT-PCR analysis of penicillin binding protein and efflux pump genes in *A. baumannii* AYE. Relative gene expression was calculated by the ΔΔ*C_T_* method, using 16S rRNA as the reference genes. (**E**) Ethidium bromide accumulation assay to determine efflux deficit state of meropenem persisters of *A. baumannii* AYE as compared to log phase cells. LOD—limit of detection. Each data represents the mean of three values and error bars indicate standard error. Significance determined by one-way ANOVA followed by Tukey’s multiple comparison test (*P < 0.01; **P < 0.001; ***P < 0.003; ****P < 0.0001; ns, non-significant).

Understanding the interplay between various genetic and cellular mechanisms is crucial for deciphering the behavior of transiently dormant antibiotic persisters. Transient dormancy in persisters is often associated with reduced metabolic activity and energy levels (32). To determine the energy state of these cells, we measured the intracellular ATP concentrations of log phase cells and meropenem persisters of *A. baumannii* AYE using a luciferase/luciferin assay (33). CCCP was used as a control, which is known to cause ATP depletion in a PMF-dependent manner (34). A significant reduction in luminescence was observed in meropenem persisters which was comparable to 1 µM ATP which was used only as a positive control (and not used as an absolute measurement) (Fig. 2A). The disruption of PMF caused due to reduction in intracellular ATP impairs energy-dependent mechanisms required for sustaining membrane potential thereby causing its perturbation (33). To test this, we used DiBAC_4_(3), an anionic lipophilic dye which enters cells with a perturbed potential state, causing enhanced fluorescence (35). We observed a significant increase in fluorescence intensity of DiBAC_4_(3) in meropenem persisters with respect to log-phase cells (Fig. 2B). This observation suggests a depolarized membrane state in meropenem persisters of *A. baumannii* (36).

The stress activation of SOS regulation of various DNA methylation and repair pathways in response to antibiotic exposure is known to temporarily halt cell growth, as observed in *E. coli* (37, 38). Such a delay in growth leads to decreased ATP consumption and altered membrane potential, which are characteristic features of persister cells (33). Predictably, we observed an increase in gene expression levels of *umuD* (central components of the SOS response), *dam* (DNA adenine methylase; involved in DNA repair), and *recA* (DNA repair) genes in meropenem persisters of *A. baumannii* (Fig. 2C) (39–41). This suggests the involvement interconnected pathways of DNA repair and metabolic regulation that lead to dormancy and survival of persisters under meropenem stress.

### Increased efflux pump activity and downregulation in genes encoding PBPs is important for persistence to meropenem stress

Accumulation of antibiotics in persisters is primarily reduced due to increased efflux activity (19). Various studies have demonstrated that RND efflux pumps are crucial for the extrusion of beta-lactam antibiotics in *A. baumannii* (42, 43). To investigate this further, we performed real-time qPCR analysis of key RND efflux pump genes, including *adeB, adeJ, adeG,* and *adeF* and observed significant overexpression in meropenem persisters as compared to log phase cells (Fig. 2D). To assess the activity of efflux pumps in persisters, we conducted an ethidium bromide accumulation (EtBr) assay. EtBr is a nucleic acid binding dye and an efflux pump substrate which accumulates inside cells with low efflux activity, resulting in increased fluorescence (44). Our results showed a decrease in EtBr fluorescence in meropenem persisters compared to log-phase cells, indicating enhanced efflux pump activity in persister cells (Fig. 2E). To further confirm the role of efflux pumps in persister formation, we utilized a deletion mutant of gene encoding *adeIJK* efflux pump in *A. baumannii* ATCC 17978. The AdeIJK efflux pump is one of the major constitutively expressed efflux systems in *A. baumannii*, with broad substrate specificity (45). The ATCC 17978 Δ*adeIJK* mutant exhibited significant defect in ability to survive meropenem exposure compared to the wild-type strain (Fig. S2A through C). Moreover, complementation of the *adeIJK* efflux pump in Δ*adeIJK* revealed 10-fold higher persister formation frequency than those of the wild-type cells (Fig. S2D). These results suggest the role of active efflux in persister cells.

Meropenem primarily targets Penicillin-Binding Protein 3 (PBP3; *ftsI*) which is essential for cell wall synthesis in *A. baumannii* (46, 47). In addition to PBP3, it also targets Penicillin-Binding Protein 1A (PBP1A; *mrcA*) and Penicillin-Binding Protein 2 (PBP2; *mrdA*) in *A. baumannii* (47). These PBPs are involved in the final stages of peptidoglycan synthesis, and their inhibition disrupts cell wall biosynthesis, leading to bacterial cell death (46). Under stress conditions, the expression of PBPs is often modulated as part of adaptive response (48). This falls in line with our observation in the real-time gene expression analysis of these major PBPs that are targeted by meropenem. We observed significant downregulation of *ftsI, mrcA,* and *mrdA* genes in meropenem persisters Fig. 2D. This indicates a survival strategy employed by persister cells that decrease the binding targets of antibiotics, thereby reducing the efficacy of the drug.

### Mechanism-based approach for identification of potential anti-persister compounds

Based on the observed characteristics of meropenem persisters in *A. baumannii*, we systematically evaluated the anti-persister potential of selected GRAS status compounds. Initially, MICs of these compounds were determined (Table S2), and those with MIC values below 1,024 µg/mL were shortlisted for further analysis. The selected compounds included carvacrol, thymol, eugenol, linalool, cinnamaldehyde, and clove oil. Subsequently, a targeted strategy utilizing fluorescence-based assays was employed to assess their efficacy in disrupting key mechanisms relevant to persisters.

**Fig 3:**
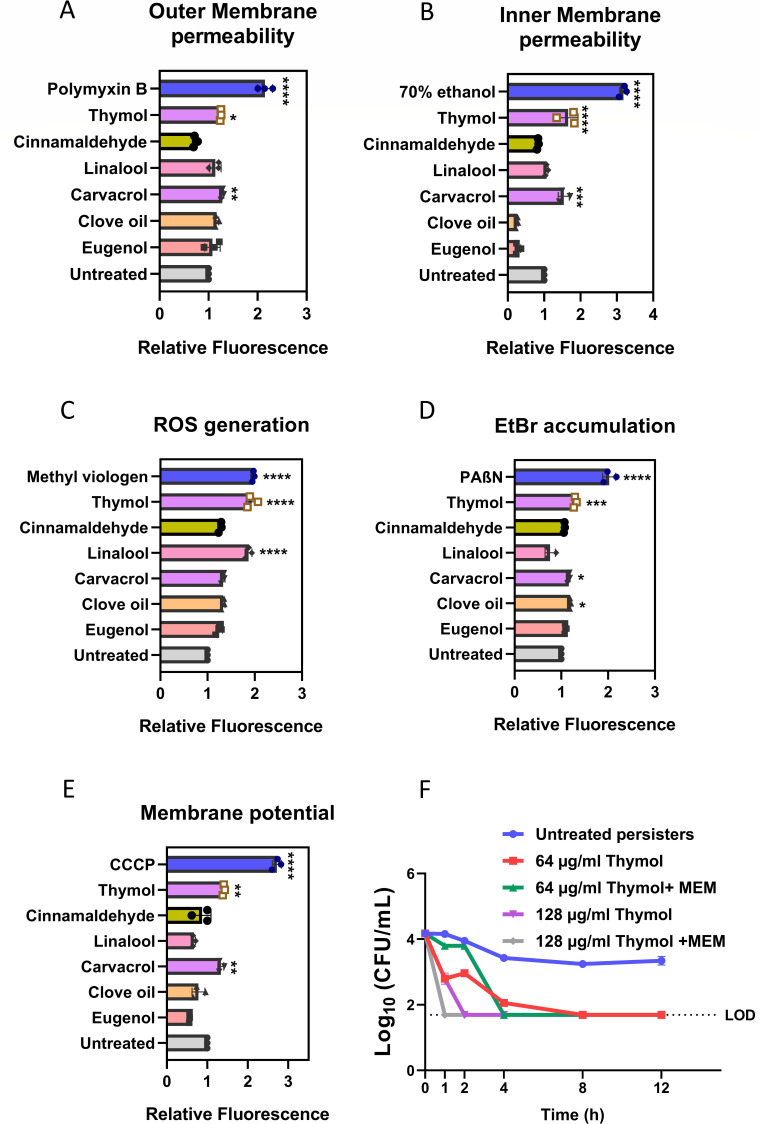
Mechanism-based assays for the identification of potential anti-persister compounds against *A. baumannii* AYE. (**A**) Outer membrane permeability assay using fluorescent probe N-phenyl-1-napthyalmine (NPN), positive control; Polymyxin B—0.25 µg/mL. (**B**) Inner membrane permeability assay using fluorescent probe Sytox Orange. Positive control—70% ethanol. (**C**) ROS generation assay using H_2_DCFDA positive control; methyl viologen 50 µM. (**D**) Efflux inhibition assay using ethidium bromide as substrate, positive control: PaßN—25 µM. (**E**) Membrane potential using fluorescent dye DiBAC_4_(3); positive control—CCCP 50 µg/mL. Relative fluorescence w.r.t. to untreated control was calculated. Thymol was chosen as the lead molecule for anti-persister studies. (**F**) Thymol exhibits bactericidal activity against meropenem persisters in *A. baumannii*. MEM—meropenem; LOD—limit of detection. Each value represents the mean of three values and error bars indicate standard error. Significance determined by one-way ANOVA followed by Tukey’s multiple comparison test (*P < 0.01; **P < 0.001; ***P < 0.003; ****P <0.0001; ns, non-significant).

The predominant strategy to eradicate persisters involves extensive disruption of the bacterial membrane (14). Therefore, the selected compounds were evaluated for their ability to induce membrane damage at sub-inhibitory concentrations using the dyes N-phenyl-1-naphthylamine (NPN) and SYTOX Orange, which are commonly employed to assess the permeability of the outer and inner membranes, respectively (49). The outer membrane permeability assay indicated that thymol, carvacrol, and linalool exhibited the highest potential to permeabilize the outer membrane of *A. baumannii* (Fig. 3A). Similarly, thymol and carvacrol demonstrated a significant ability to compromise the inner membrane integrity (Fig. 3B). Bacterial cells generate ROS, such as superoxide and hydrogen peroxide, in response to various stresses and as metabolic by-products (50). To mitigate cellular damage caused by ROS, bacteria activate an oxidative stress response via antioxidant enzymes (51). The active suppression of oxidative stress and reduction in ROS production are known mechanisms contributing to antibiotic tolerance in bacteria (50). Thus, evaluating for compounds with pro-oxidant properties represents another effective strategy to target persisters. The potential of the compounds to enhance ROS production was assessed using dichloro-dihydro-fluorescein diacetate (H_2_DCFDA) (52). The results indicated that linalool and thymol acted as pro-oxidants in *A. baumannii* cells (Fig. 3C), thereby suggesting their potential as ROS-generating anti-persister agents. As discussed previously, active efflux mechanisms have been established to contribute significantly to antibiotic tolerance in bacteria (43). Therefore, administration of efflux pump inhibitors (EPI) in combination with antibiotics is an important strategy to target the persister phenotype (53). Hence, these compounds were further screened for their potential to inhibit efflux of EtBr (54), a substrate for most efflux pumps in gram-negative bacteria. The assay revealed thymol to be the most potent EPI, followed by clove oil, linalool, and carvacrol (Fig. 3D). We also observed the activity of these compounds to dissipate the proton motive force since most efflux pumps are driven by it (43). To evaluate this, we used the fluorescent dye DiBAC_4_(3) to assess the impact of various compounds on *A. baumannii* membrane potential (36). Thymol and carvacrol were found to cause the greatest membrane depolarization (Fig. 3E). These lead compounds were further evaluated for their ability to effectively eradicate *A. baumannii* persisters.

### Thymol treatment eradicates *A. baumannii* persisters

The anti-persister potential of three lead compounds thymol, carvacrol, and linalool obtained from the mechanistic assays was evaluated. Persister cells isolated post exposure to meropenem were subsequently incubated with the lead compounds for 12 h, with or without meropenem stress. The efficacy of the treatment was assessed by determining the surviving fraction, compared to untreated persister cells. As observed from the time-kill kinetics assay, thymol alone achieved complete eradication of meropenem persisters of *A. baumannii* at 64 µg/mL (0.25× MIC) in 8 h post treatment (Fig. 3F). Notably, when combined with meropenem, thymol was able to completely eradicate persisters at a concentration of 128 µg/mL (1× MIC) in 4 h post treatment. Linalool and carvacrol also demonstrated inhibitory effects on *A. baumannii* persisters, at higher concentrations (Fig. S3). Interestingly, thymol exhibited superior anti-persister activity compared to carvacrol despite both being structural isomers. The enhanced activity of thymol at comparatively lower concentrations than carvacrol can be attributed to its superior membrane perturbing activity as evident from the mechanistic assay.

**Fig 4:**
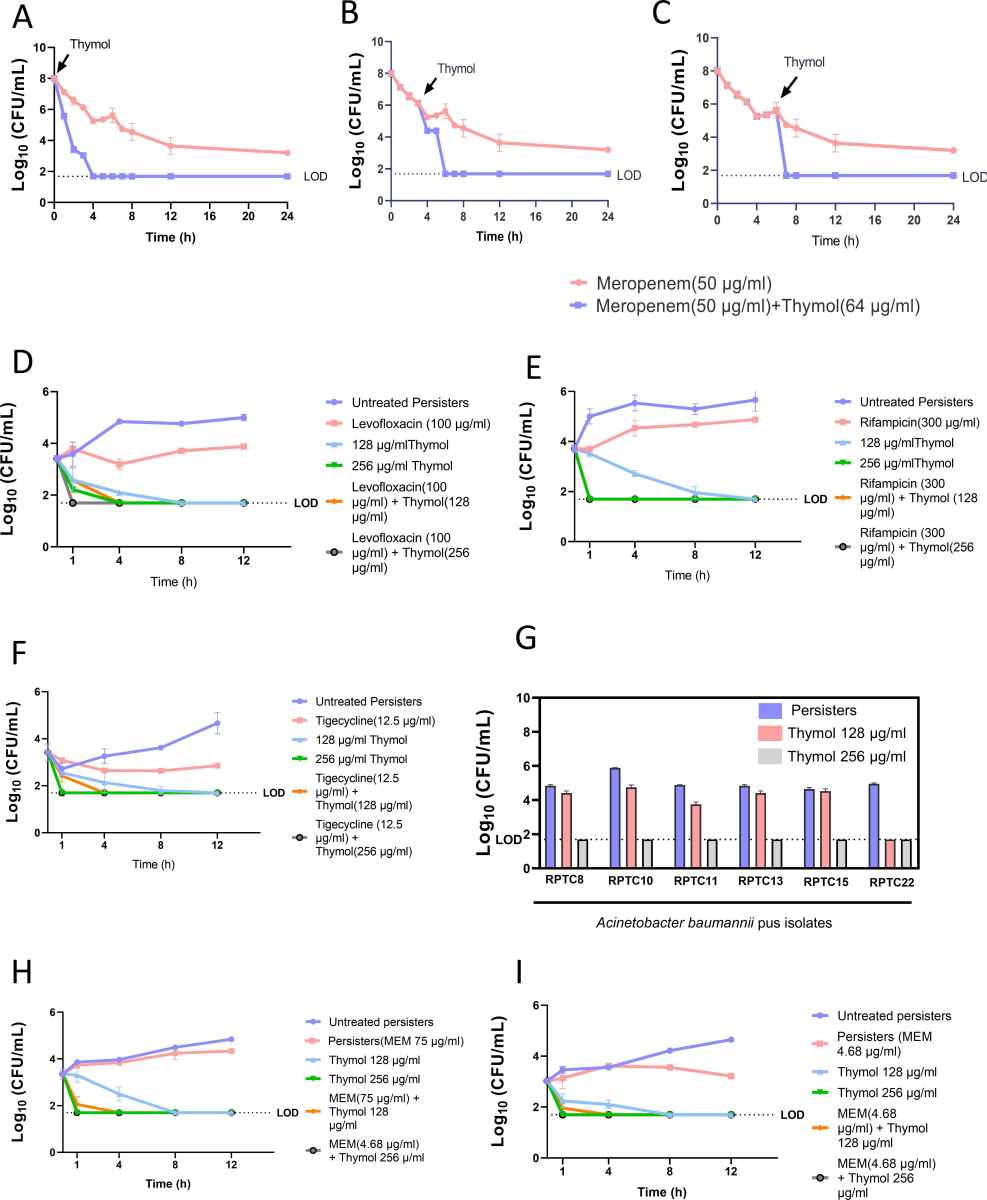
Anti-persister activity of thymol is time and antibiotic independent and ranges over various clinical strains: (**A–C**) Log phase cells of *A. baumannii* AYE were treated with 50 µg/mL of meropenem and 64 µg/mL of thymol was added at time points (**A**) *t* = 0 h, (**B**) 3 h, and (**C**) 6 h as indicated by arrows. Surviving persisters were enumerated by plating. Each value represents the mean of three values and error bars indicate standard error. (**D–F**) Kill kinetic of (**D**) levofloxacin, (**E**) rifampicin, and (**F**) tigecycline persisters of *A. baumannii* AYE were treated with various concentration of thymol alone and in the presence of respective antibiotics. (**G**) Meropenem persisters (shown in blue) from six clinical isolates were exposed to thymol. Viable counts were determined after 12 h incubation. Each bar represents the mean of three values and error bars indicate standard error. (**H and I**) Kill kinetics of meropenem persisters of (**H**) *Klebsiella pneumoniae* ATCC 700698 and (**I**) *Pseudomonas aeruginosa* MTCC 2543 treated with thymol alone and in the presence of meropenem stress. LOD—limit of detection. Each value represents the mean of three values and error bars indicate standard error. Significance determined by one-way ANOVA followed by Tukey’s multiple comparison test (*P < 0.01; **P < 0.001; ***P < 0.003; ****P <0.0001; ns, non-significant).

We investigated the impact of thymol administration at different time points on the formation of a persister population upon meropenem treatment. Our objective was to determine whether thymol could exert its effects earlier in the treatment process, potentially preventing the establishment of persisters. To this end, thymol was added at various time points during meropenem treatment of log-phase *A. baumannii* AYE cells. Remarkably, thymol was able to completely eradicate the bacterial population within 1–3 h of treatment, irrespective of the timing of its addition (Fig. 4A through C). These findings suggest that thymol effectively prevents the formation of persisters by intervening early in the treatment process, even before the transition to the second phase of the biphasic kill curve.

We also evaluated whether the anti-persister activity of thymol depended on the type of antibiotic used to induce persistence. Persisters of *A. baumannii* AYE upon rifampicin, tigecycline, and levofloxacin exposure were isolated (Fig. S1A through C) and treated with thymol, both as monotherapy and in combination with the respective antibiotics. Thymol completely eradicated persisters of all three antibiotics at 128 µg/mL within 8 h post treatment (Fig. 4D through F). These findings clearly demonstrate that thymol possesses antibiotic-independent anti-persister activity and can be effectively combined with various antibiotic classes.

The findings obtained so far were further validated by evaluating the activity of thymol against a collection of in-house multidrug-resistant (MDR) clinical isolates of *A. baumannii*. Log phase cultures of *A. baumannii* clinical strains were exposed to bactericidal concentrations meropenem for 24 h to isolate the surviving persister fractions. All clinical strains displayed a typical biphasic growth pattern upon treatment with meropenem (Fig. S1F through K). Thymol treatment significantly inhibited meropenem persisters of MDR *A. baumannii* clinical isolates, both as monotherapy and in combination with meropenem (Fig. 4G).

We also tested thymol against other gram-negative ESKAPE pathogens, specifically *K. pneumoniae* and *P. aeruginosa*. Meropenem persisters were first isolated for *K. pneumoniae* ATCC 700698 and *P. aeruginosa* MTCC 2543, confirming the presence of persister cells in these strains (Fig. S1D and E). Thymol treatment completely eradicated *K. pneumoniae* persisters at 128 µg/mL (0.25× MIC) (Fig. 4H) and eliminated *P. aeruginosa* persisters at a similar concentration (0.125× MIC) (Fig. 4I). These results underscore the potential of thymol as an effective anti-persister agent against multiple gram-negative pathogens.

### Thymol has multifaceted activity that targets several key survival mechanisms of meropenem persisters

**Fig 5:**
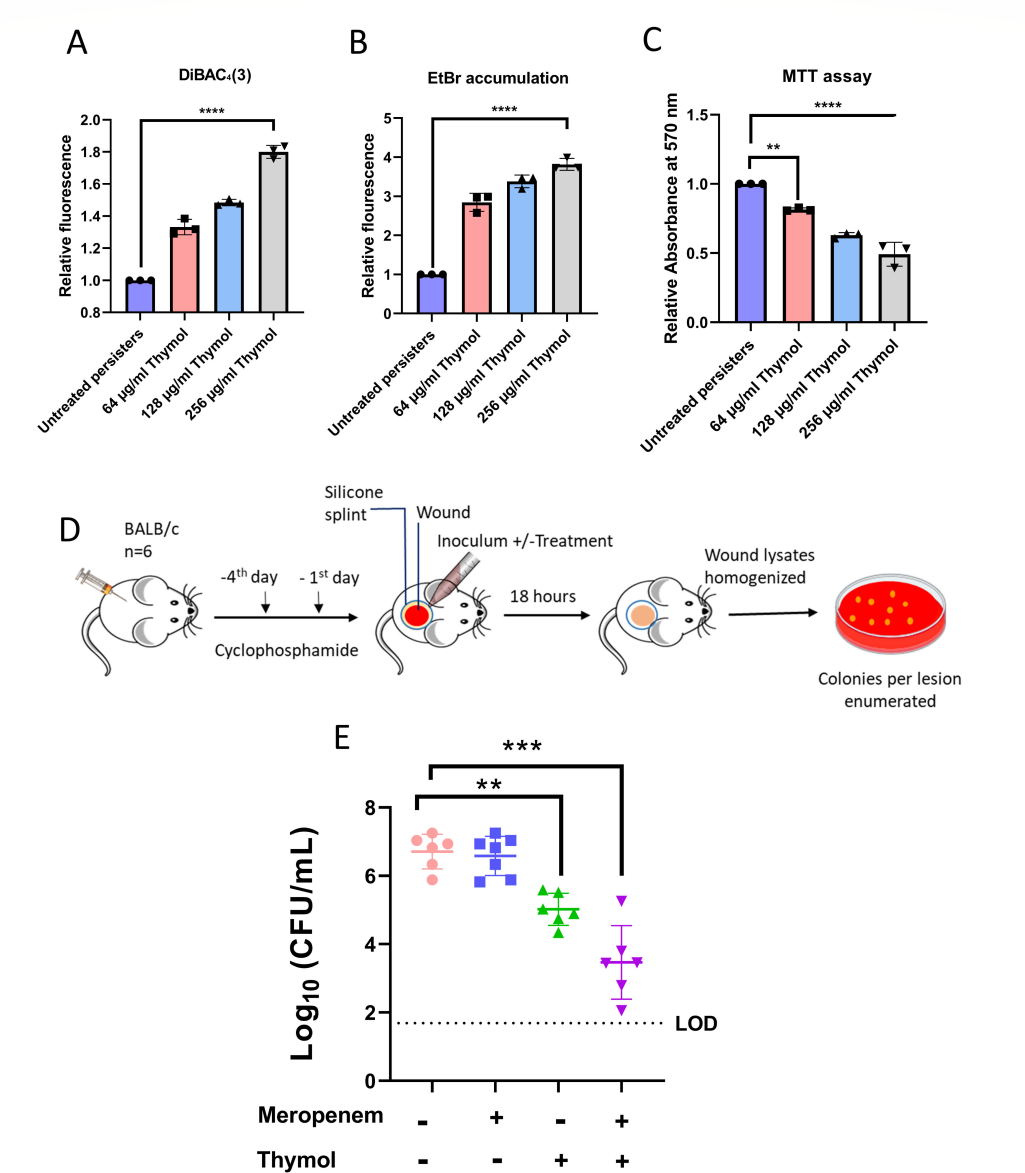
(**A**) Effect of thymol on membrane potential of meropenem persisters *of A. baumannii* AYE. Relative increase in DiBAC_4_(3) fluorescence in the presence of thymol with respect to untreated persisters was calculated. (**B**) Ethidium bromide (EtBr) accumulation assay in meropenem persisters. Relative fluorescence of EtBr accumulation upon the addition of thymol with respect to untreated persisters was calculated. (**C**) Thymol inhibits the metabolic activity of meropenem persisters of *A. baumannii* AYE in the MTT assay. Relative absorbance upon thymol treatment with respect to untreated persisters was calculated. Each value represents the mean of three values, and error bars indicate standard error. Significance determined by one-way ANOVA followed by Tukey’s multiple comparison test. (**D**) Schematics of mouse wound infection model. Mice (*n* = 6 per group) were infected persisters of *A. baumannii* AYE. (**E**) Thymol treatment enhances the eradication of meropenem persisters of *A. baumannii* in a murine wound infection model. To evaluate the survival of these persisters*, A. baumannii* cells upon meropenem treatment were inoculated into wounds on mice, followed by thymol treatment. Wound lysates were then plated to assess the effectiveness of thymol in eliminating *A. baumannii* persister cells in-vivo. LOD—limit of detection. Each value represents the mean of three values and error bars indicate standard error. Significance determined by one-way ANOVA followed by Tukey’s multiple comparison test (*P < 0.01; **P < 0.001; ***P < 0.003; ****P <0.0001; ns, non-significant).

To validate the mechanistic strategy employed based on the activity of GRAS compounds against log phase *A. baumannii* cells, we examined similar effects on *A. baumannii* persisters. To determine the mechanism by which thymol eradicates persister cells, we first examined its effect on the membrane potential of *A. baumannii* AYE persisters. From the mechanistic study, we observed a depolarised membrane state upon treatment with bactericidal concentration of meropenem (Fig. 2B), which can be attributed to a stress response mechanism resulting from disruption of energy state and ionic balance. Upon treatment with thymol, the persister cells exhibited a concentration dependent increase in fluorescence of DiBAC_4_(3) suggesting further perturbation of bacterial membrane potential (Fig. 5A). Thymol is known for its ability to disrupt cell membrane and perturb ionic balance (55). When used in combination with antibiotic stressor such as meropenem, the impact of thymol on membrane potential can be more pronounced. Thymol exacerbates the existing membrane damage caused by the antibiotic leading to further depolarisation.

The increase in efflux pump activity was observed to be a key survival mechanism of persisters upon meropenem treatment as observed from the EtBr accumulation assay and gene expression analysis (Fig. 2D and E). We observed a concentration-dependent increase in EtBr accumulation when persisters were treated with thymol (Fig. 5B), which could be attributed to the ability of thymol to inhibit efflux pump activity and its ability to increase membrane permeability (56). These dual effects suggest that thymol may disrupt the membrane integrity of bacterial cells, leading to enhanced EtBr accumulation.

The metabolic inhibitory activity of thymol has been previously demonstrated where it significantly reduces the viability of *E. coli* and *S. aureus* (55). Consequently, we performed an MTT assay to evaluate the effect of thymol on the metabolic activity of meropenem persisters (57). Thymol treatment resulted in a significant reduction in formazan crystal formation, indicating decreased metabolic activity in the persisters and underscoring its efficacy as an anti-persister agent (Fig. 5C).

### Thymol treatment enhances killing of meropenem persisters of *A. baumannii* in a murine wound infection model

To provide a proof-of-principle for *in-vivo* efficacy of thymol against persister cells, we utilized a murine acute wound infection model as described in the schematic (Fig. 5D) (31). Consequently, meropenem persisters of *A. baumannii* AYE (concentrated to 10^6^ CFU/mL) were seeded on the dorsal wound. Subsequently, we administered cell-free medium control, cell-free medium with meropenem to maintain antibiotic pressure, cell-free media with thymol alone, and cell-free medium with a combination of meropenem and thymol. The quantitative analysis revealed that the presence of meropenem alone did not result in the reduction of bacterial load as compared to cell free media. In contrast, treatment with thymol alone revealed a 1-log_10_ fold reduction in the number of colonies of persisters. Notably, when thymol was used in combination with meropenem more than 3-log_10_ fold reduction in colonies was observed (Fig. 5E). These results highlight that persister cells mechanistically adapt to high concentrations of meropenem alone, emphasizing the need for additional treatments like thymol. Our findings support the potential clinical use of thymol in treating infections involving transient persister population.

## DISCUSSION

Persister cells are a subpopulation of bacteria that can survive antibiotic treatment without acquiring genetic resistance, thus leading to treatment failure and relapse once antibiotic pressure is removed (26). In this study, the characteristics of spontaneous persister formation in *A. baumannii* were determined following treatment with one of the last-resort antibiotic, meropenem. Previous studies have shown that spontaneous *E. coli* persisters in the log phase exhibit multi-drug tolerance and reduced permeability when exposed to ciprofloxacin (58), while *P. aeruginosa* persisters upon ceftazidime exposure display high redox activity and elevated stringent response (59). These studies emphasize the common mechanisms of reduced permeability, decreased membrane potential, and metabolic downregulation in persister formation across different bacterial species.

Consequently, we wanted to assess if the mechanism adopted by spontaneous persisters formed by treating log phase cells of *A. baumannii* with meropenem. We deciphered the involvement of intracellular ATP and membrane permeability in maintaining meropenem persistence. Membrane potential (PMF), the product of cellular respiration, describes the electrochemical gradient across the cytoplasmic membrane, composed of an electrical gradient (Δ*ψ*) and a chemical gradient (ΔpH) (60). This potential is crucial for cellular energy production (61). Meropenem persisters exhibited compromised membrane permeability due to disruption of the Δ*ψ* component as observed from the depolarized state in the DiBAC_4_(3) assay. This disruption leads to the conversion of the *F*_0_-*F*_1_ ATP synthase complex into an ATP-driven proton pump (62), causing a shift to a non-growing phenotype by depleting intracellular ATP. Additionally, we observed an increase in drug efflux activity in these persisters. This enhanced efflux activity is consistent with findings from other studies where persisters exhibited increased efflux pump activity as a survival mechanism (53). Our findings also revealed a reduction in the expression of various PBPs, which are the molecular targets of meropenem. This observation is consistent with literature that indicate decreased expression of various important classes of PBPs are a common mechanism by which bacteria evade the effects of β-lactam antibiotics. Previous studies have highlighted how modifications in PBPs can lead to reduced susceptibility to β-lactams in *E. coli* (63) and reduced PBP expression contributes to antibiotic tolerance in *S. aureus* (64). Together, these findings suggest that spontaneous persister formation in *A. baumannii* upon meropenem treatment shares common persistence mechanisms with other pathogens, including enhanced efflux pump activity and target modification.

The discovery of novel agents capable of eradicating bacterial persisters has been significantly hampered by the limitations of current time-consuming anti-persister assays (31). To address this, we utilized a strategy aimed at identifying potential anti-persister candidates based on their ability to perturb these persistence mechanisms. This strategy led us to identify thymol, as an effective anti-persister agent against *A. baumannii*. Thymol (2-isopropyl-5-methylphenol) is a monoterpene phenol derived from plants in the Lamiaceae family and can also be synthesized chemically (65). It is widely recognized for its safety, having received GRAS (Generally Recognized as Safe) status from the FDA (21CFR172.515), with an LD_50_ of 4,000 mg/kg in rat models, indicating low toxicity upon oral administrations (66). Thymol exhibits antibacterial activity against both gram-negative and gram-positive pathogens, including *E. coli*, *Salmonella Typhimurium*, *Proteus mirabilis*, and *Listeria innocua* (67). Although few studies have explored the activity of GRAS compounds against *A. baumannii* (68, 69), none have evaluated its efficacy against persister cells. Our study demonstrates potent inhibitory activity of thymol against spontaneous *A. baumannii* persisters isolated in the presence of meropenem and other antibiotics. The anti-persister activity of thymol also encompasses other gram-negative pathogens including *K. pneumonieae* and *P. aeruginosa*. Previous studies have demonstrated the ability of thymol to permeabilize cell membranes and effectively disrupt biofilms in *S. aureus* and *S. epidermidis* (70). Additionally, thymol has been shown to generate ROS and inhibit efflux pumps, thereby enhancing its antibacterial efficacy against a range of pathogens (65). It is well established that persister cells are characterized by dormancy and metabolic inactivity that contribute to their ability to survive antibiotic treatment (71). Inhibiting their already low metabolic functions further can be significant in disrupting their survival mechanisms (71). Our data also align with evidence that thymol disrupts bacterial membranes, reduces membrane potential, and inhibits ATP synthesis, resulting in decreased metabolic activity and increased eradication of persisters. However, none of the studies evaluated the inhibitory potential of thymol against persisters of *A. baumannii or* other ESKAPE pathogens. Moreover, our data suggest that thymol could eradicate meropenem persisters of *A. baumannii* in a murine wound infection model. These results imply that thymol has significant clinical applications for treating persistent infections. Our findings align with existing research that highlights the efficacy of thymol against a range of bacterial pathogens, suggesting that targeting the bacterial membrane could be a viable strategy for combating persistent infections.

## MATERIALS AND METHODS

### Bacterial strains, culture conditions, and reagents

The strains *A. baumannii* AYE, *K. pneumoniae* ATCC 700698, and *P. aeruginosa* MTCC 2453 were used for the study. The growth medium was Cation adjusted Muller Hinton Broth (CAMHB), Luria Bertani (LB) broth, LB agar, or Leeds Acinetobacter agar. Antibiotics and GRAS compounds used in this study were procured from Sigma-Aldrich and Tokyo Chemicals Industries Limited (TCI) and stored as prescribed. Dichloro-dihydro-fluorescein diacetate (H_2_DCFDA), DiBAC_4_(3), NPN, and SYTOX orange were purchased from Thermo Fisher Scientific. *A. baumannii* clinical isolates were procured from AIIMS, Bhopal and Government Medical College and Hospital, Chandigarh, India.

### Determination of minimum inhibitory concentration

Serial twofold dilutions of each compound were prepared in CAMHB in 96-well plates, as per CLSI guidelines (72). Overnight grown cultures of test strains were sub-cultured and incubated at 37°C and 180 RPM till they reached an optical density of 0.5 McFarland. Cultures were subsequently diluted to achieve inoculum density of ~10^5^ CFU/mL and added to plates containing the compound dilutions. Plates were incubated for 16 h at 37°C in a static incubator. At the end of incubation, absorbance was recorded on a plate reader (SpectraMaxM2e) at 600 nm. The minimum concentration at which no growth was observed was considered the MIC.

### Persister assay

A single colony of *A. baumannii* AYE was cultured at 37°C for 16  h in LB, subcultured to 1:100 in fresh medium, and incubated until mid-log phase (0.5 OD_600_). The minimum concentration of antibiotics was determined at which a biphasic kill curve is observed. These culture were treated with meropenem (50 µg/mL), tigecycline (12.5 µg/mL), rifampicin (300 µg/mL), and levofloxacin (150 µg/mL) and incubated for 12 hours at 37°C at 180 rpm. For the perister assay with wild-type *A. baumannii* ATCC 17978, ATCC 17978 Δ*adeIJK*, and ATCC 17978 *adeIJK::adeIJK* strains, meropenem at a concentration of 150 µg/mL was used. Expression of *AdeIJK* efflux pump in ATCC 17978 adeIJK::adeIJK strain was induced by adding 1 mM IPTG to fresh media, at the time of sub-culturing (73).

Post incubation, cells were harvested, serially diluted, and spotted on LB agar plates for colony counts. A single colony was re-inoculated into fresh LB medium, and an *in vitro* susceptibility experiment was performed to ensure the MIC had not changed in order to confirm that the surviving colonies were persister cells. Relative frequency of persister formation to different antibiotics w.r.t. log phase cells was calculated as number of colonies obtained after treatment/number of colonies obtained in untreated log phase cells.

Persister cells surviving the antibiotic treatment up to 24 h were harvested, washed with 1× PBS, and regrown for 24 h at 37°C in LB broth without antibiotics. The culture was diluted 1:100 with fresh LB broth to obtain log phase cells (O.D. 0.5) and again treated with the respective antibiotics up to 24 h. This cycle was repeated three times and antibiotic-treated cells from each passage were withdrawn at different time intervals (0, 2, 4, 8, 24 h) to determine the number of surviving persister cells. Similar persister assay was followed for all other strains used in this study.

### ATP quantification assay

*A. baumannii* AYE was grown till 0.5 OD_600_ and treated with meropenem (50 µg/mL) for 12 h at 37°C. Cells were washed thrice with 1× PBS, specifically to remove dead and damaged cells from the population. The isolated persister population was concentrated to 0.5 OD_600_ (10^7^ CFU/mL) and resuspended in 1× PBS, to maintain equal number of cells in both treated and control groups for accurate comparison. Intracellular ATP levels were measured using the BacTiter-Glo kit according to the manufacturer’s protocols. The background ATP level was subtracted from spent medium from each sample, and 1 µM ATP was used as the positive control. Luminescence was monitored in 96-well white plate using a Synergy H1 plate reader.

### Assay for measurement of membrane potential

Isolated persister cells re-suspended in 1× PBS and exposed to 5 µM DiBAC_4_(3) followed by incubation for 30 min at room temperature. Fluorescence readings were taken at excitation and emission wavelengths of 485 nm and 528 nm, respectively, in 96-well black plates using a Synergy H1 plate reader. The CFU/mL of the meropenem-treated group was adjusted to 0.5 OD_600_, corresponding to 10⁷ CFU/mL, to ensure an equal number of cells in both the treated and control groups for accurate comparison. Isolated persisters were treated with thymol at 0.5×, 1×, and 2× MIC and incubated for 1 h at 37°C. The ability of thymol to further depolarize membrane of meropenem persisters of *A. baumannii* was also measured using a similar protocol.

### Quantitative real-time PCR

Briefly, 1 µg of total isolated RNA was used to prepare cDNA using the SuperScript III first-strand synthesis kit (Invitrogen). q-Real-time PCR was performed with the SYBR green master mix (Applied Biosystems) following the manufacturer’s instructions. Measurements were performed using the QuantStudio 6× real-time PCR system (Applied Biosystems) with the following conditions: 95°C for 10 min, 40 cycles of 95°C for 15 s, and 60°C for 1 min, and a final dissociation cycle of 95°C for 2 min, 60°C for 15 s, and 95°C for 15 s. Relative gene expression was calculated by the ΔΔCt method using 16S rRNA as the reference gene. Experiments were performed in biological duplicates and measured in technical triplicates. Primers used for real-time PCR are given in Table S4.

### Mechanism-based assays

For all assays, 0.25× MIC (Table S2) of the compounds eugenol, clove oil, carvacrol, linalool, cinnamaldehyde, and thymol were used. Overnight cultures of *A. baumannii* AYE were sub-cultured in fresh medium and incubated at 37°C and 180 RPM until OD_600_ reached 0.5. Cells were washed and resuspended in 5 mM HEPES or 1× PBS buffer to an OD_600_ of 0.3. The details of the assays are provided in the supplemental methods.

### Anti-persister activity and kill kinetics assays

Persisters of *A. baumannii* AYE, *K. pneumoniae* ATCC 700698, *P. aeruginosa* MTCC 2453, and other clinical strains were isolated by treating the log phase cells with meropenem or other antibiotics (rifampicin, levofloxacin, and tigecycline) as described earlier. Persister cells were incubated with varying concentrations of test compounds, thymol, linalool, and carvacrol with or without antibiotics, at 37°C, 180 rpm. The number of viable cells was enumerated at various time points for 12 h.

The kinetics of inhibition of *A. baumannii* in the presence of meropenem (at 50 µg/mL) upon addition of thymol was studied. Briefly, log phase cells of *A. baumannii* AYE were treated with meropenem or combination of meropenem and 1× MIC of thymol. Thymol was added to the cultures at different time points *t* = 0 h, 3 h, and 6 h. The number of viable cells was determined at regular intervals in terms of CFU/mL.

### MTT assay

Meropenem persisters of *A. baumannii* AYE were washed thrice with 1× PBS and resuspended in the same buffer. Isolated persisters were treated with thymol at 0.5×, 1×, and 2× MIC and incubated for 1 h at 37°C MTT dye (0.5 mg/mL) was added to the cells and incubated for 2 h followed by addition of DMSO. Absorbance was read at 570 nm to assess the reduction of MTT dye and the formation of soluble formazan. Relative absorbance was calculated with respect to the untreated control group.

### *In vivo* murine infection model

The details of the *in vivo* murine infection are provided in the supplemental material.

## SUPPLEMENTAL MATERIAL

Supplemental material aac.01381-24-s0001.docx Figures S1 to S3, Tables S1 to S4, and supplemental methods.

ASM does not own the copyrights to Supplemental Material that may be linked to, or accessed through, an article. The authors have granted ASM a non-exclusive, world-wide license to publish the Supplemental Material files. Please contact the corresponding author directly for reuse.
