# Supplementary material for: Deciphering meropenem persistence in Acinetobacter baumannii facilitates discovery of anti-persister activity of thymol
Source: Antimicrob Agents Chemother. 2025 Feb 20;69(4):e01381-24. doi: 10.1128/aac.01381-24 (PMC11963602; doi:10.1128/aac.01381-24)
Supplement: Supplemental material — Figures S1 to S3, Tables S1 to S4, and supplemental methods. [file aac.01381-24-s0001.docx]

**Deciphering Meropenem Persistence in *Acinetobacter baumannii* Facilitates Discovery of Anti-persister Activity of Thymol**

Arsalan Hussain^1,^*, Timsy Bhando^1,^*^,a^, Ananth Casius^1,^*^,b^, Rinki Gupta^1^, Ranjana Pathania^1,#^

^1^Department of Biosciences and Bioengineering, Indian Institute of Technology Roorkee, Roorkee 247667, India

Running head: Targeting spontaneous persistence in *A. baumannii.*

^#^ Address correspondence to Ranjana Pathania, [ranjana.pathania@bt.iitr.ac.in](mailto:ranjana.pathania@bt.iitr.ac.in)

*Contributed equally

^a^Present address:, Michael G. DeGroote Institute of Infectious Disease Research, McMaster University, Hamilton, Canada

^b^Present address:, Department of Microbiology, Center for RNA Biology, The Ohio State University, Columbus, Ohio 43210, USA.

**Supplementary Figures: S1-S3**

**Supplementary Tables: S1-S4**

**Supplementary Methods**


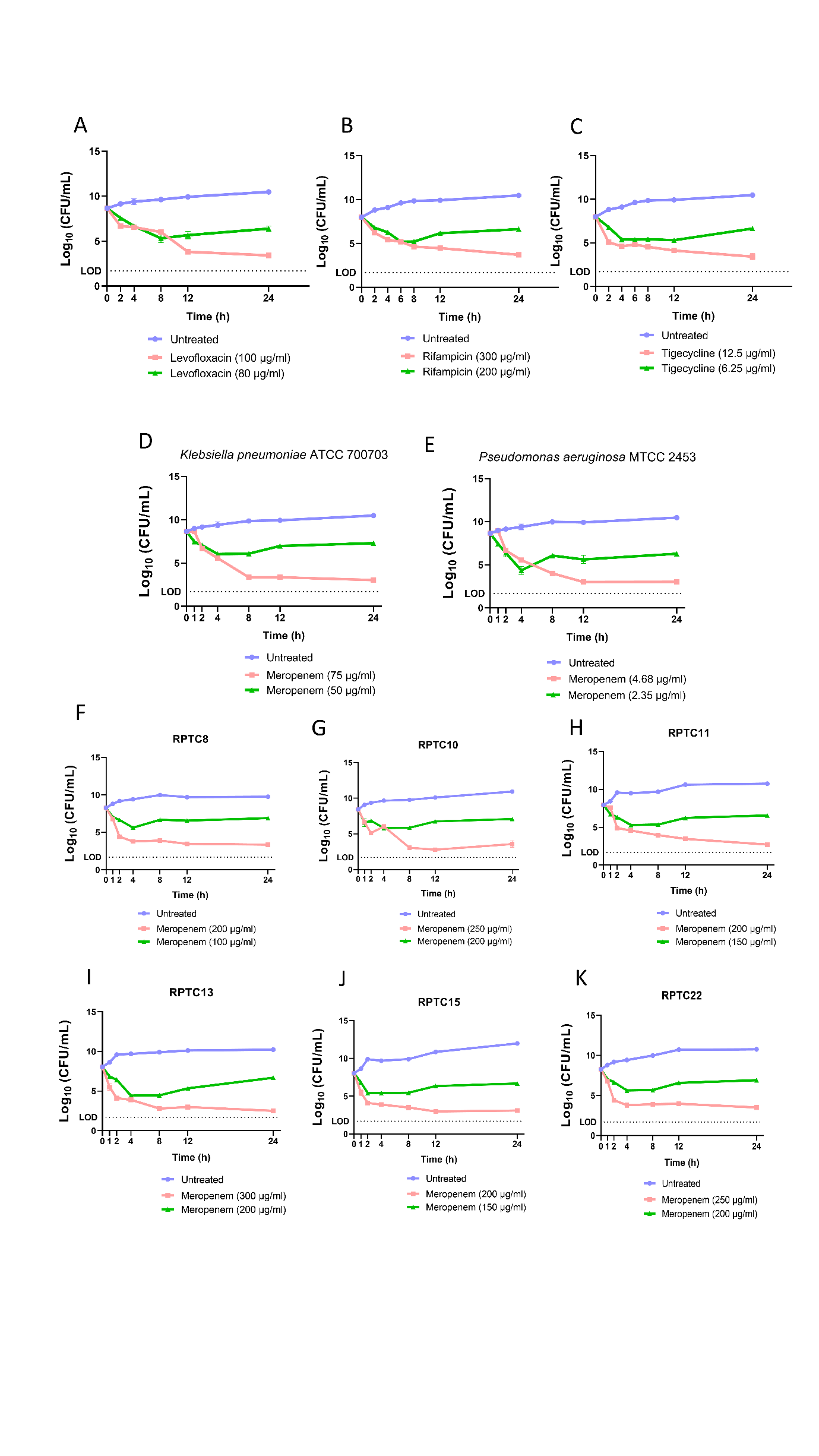


**Figure S1:** Minimum concentration at which biphasic killing pattern observed **(A-C)** Biphasic kill pattern of *A. baumannii* AYE treated with (A) Levofloxacin, (B) Rifampicin and (C) Tigecycline**. (D-E)** Biphasic kill pattern of (D) *K. pneumoniae* and (E) *P. aeruginosa* treated with meropenem. **(F-K)** Biphasic kill pattern observed in clinical strains of *A. baumannii*. Each value represents the mean of three values and error bars indicate standard error.


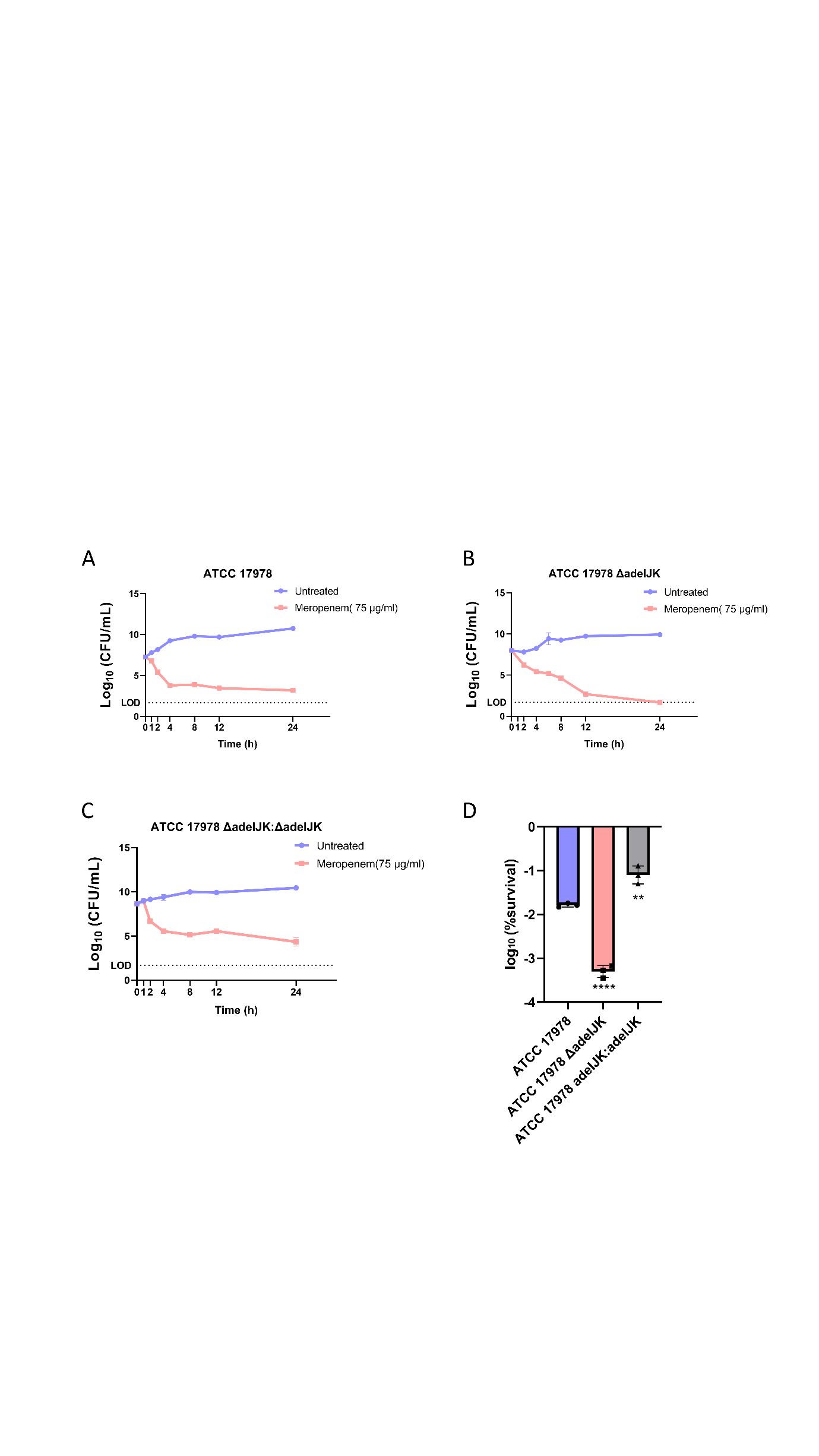


**Figure S2: (A-C)** Biphasic kill pattern of (A) *A. baumannii* ATCC17978, (B) Δ*adeIJK*, and (C) *adeIJK:adeIJK* treated with meropenem at concentration of 75 μg/ml. **(D)** Comparative percentage survival rate of *A. baumannii* ATCC17978 , mutant and complementation strain upon meropenem treatment at 75 μg/ml after 12 hours of incubation. Each value represents the mean of three values and error bars indicate standard error. Significance determined by one-way ANOVA followed by Tukey’s multiple comparison test. (**, P<0.01; ***, P<0.0001; ns, non-significant).


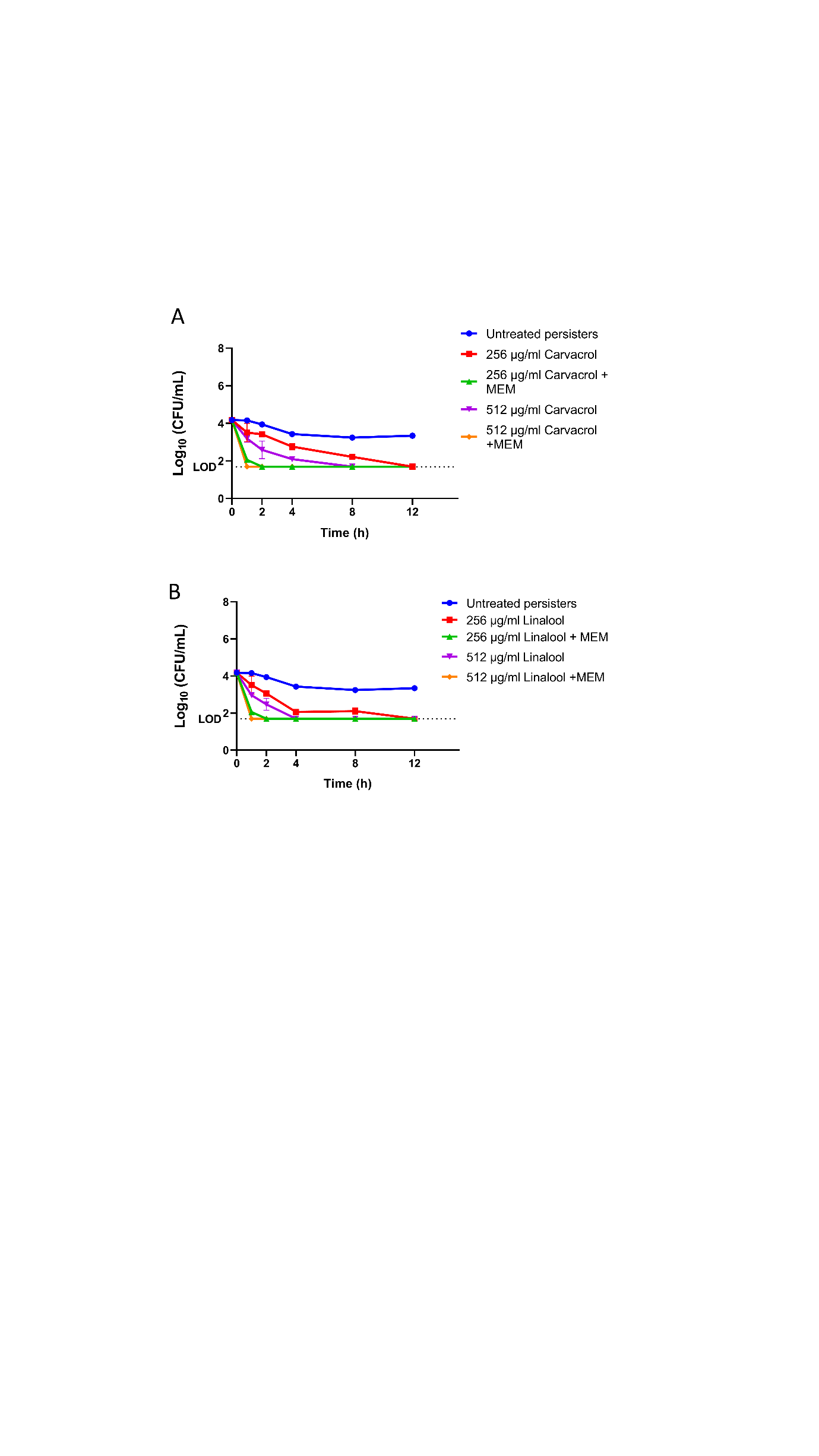


**Figure S3:** Anti-persister activity of (A) Carvacrol and (B) Linalool against meropenem persisters of *A. baumannii* AYE. Each value represents the mean of three values and error bars indicate standard error.

**SUPPLEMENTARY TABLES:**

**Table S1: List of strains used in this study**

| **Bacterial isolate** | **Isolate number** | **Source** |
| --- | --- | --- |
| *A. baumannii* | AYE | Purchased from ATCC Manassas, USA |
| *A. baumannii* | ATCC 17978 | Purchased from ATCC Manassas, USA |
| *A. baumannii* | RPTC 8 | Lab collection(1) |
| *A. baumannii* | RPTC 10 | Lab collection(1) |
| *A. baumannii* | RPTC 11 | Lab collection(1) |
| *A. baumannii* | RPTC 13 | Lab collection(1) |
| *A. baumannii* | RPTC 15 | Lab collection(1) |
| *A. baumannii* | RPTC 22 | Lab collection(1) |
| *A. baumannii* | ATCC 17978 Δ*adeIJK* | Gift from Prof. Ayush Kumar; University of Manitoba |
| *A. baumannii* | ATCC 17978 *adeIJK:adeIJK*  (Complementation was performed using the pUC18T-mini-Tn7T-LAC-Gm vector, which integrates the *adeIJK* genes into a neutral genomic site on the A. baumannii chromosome downstream of the *glmS* gene (2)). | Gift from Prof. Ayush Kumar; University of Manitoba (2). |
| *P. aeruginosa* | MTCC 2453 | Purchased from MTCC, Chandigarh, India |
| *K. pneumoniae* | ATCC 700603 | Purchased from ATCC, Manassas, USA |

**Table S2: Minimum Inhibitory Concentration (MIC) of GRAS compounds and other antibiotics against *A. baumannii* AYE**

| **Compounds** | **MIC (μg/ml)** |
| --- | --- |
|  |  |
| Carvacrol | 128 |
|  |  |
| Cinnamladehyde | 256 |
|  |  |
| Clove Oil | 512 |
|  |  |
| Eugenol | 512 |
|  |  |
| Thymol | 256 |
|  |  |
| Linalool | 512 |
|  |  |
| Origanum oil | 1024 |
| Curcumin | >1024 |
| Berberine | >1024 |
| Chlorogenic acid | >1024 |
| Piperine | >1024 |
|  |  |
| Meropenem | 0.5 |
|  |  |
| Rifampicin | 4 |
|  |  |
| Tigecycline | 0.25 |
|  |  |
| Levofloxacin | 4 |
|  |  |
| Polymyxin B | 0.25 |

**Table S3: MIC values of meropenem and thymol against various Gram negative strains.**

| **Strain No:** | **Meropenem (μg/ml)** | **Thymol (μg/ml)** |
| --- | --- | --- |
|  |  |  |
| *A. baumannii* RPTC8 | 4 | 512 |
|  |  |  |
| *A. baumannii* RPTC10 | 8 | 512 |
|  |  |  |
| *A. baumannii* RPTC11 | 2 | 256 |
|  |  |  |
| *A. baumannii* RPTC13 | 4 | 512 |
|  |  |  |
| *A. baumannii* RPTC15 | 8 | 512 |
|  |  |  |
| *A. baumannii* RPTC22 | 8 | 512 |
|  |  |  |
| *K. pneumoniae* ATCC 700703 | 0.5 | 512 |
|  |  |  |
| *P. aeruginosa* MTCC 2453 | 0.0315 | 1024 |
| *A. baumannii* ATCC 17978 | 0.25 | 512 |

**Table S4: List of q-RT PCR primers used in the study.**

|  | Gene | Primers | Sequence |
| --- | --- | --- | --- |
| 1 | Dam | Forward | TAGTGTCGGGTGGAGGTAGG |
| 2 | Dam | Reverse | CGCTGCTGAAATGTGGTGTG |
| 3 | UmuD | Forward | GGTCACTACTCCCCAAATCACC |
| 4 | umuD | Reverse | TGATACCCAATTTCAGCCCCC |
| 5 | recA | Forward | GAGTCACCCATCTCACCTTCG |
| 6 | recA | Reverse | CGACAATGGTGAGCAAGCAC |
| 7 | phoU | Forward | GGTATGGCGTGCATCAAAGC |
| 8 | phoU | Reverse | CGCGTCATATTGCTCGTGTG |
| 9 | glpD | Forward | GGGTGTGCGTGCTTTATGTG |
| 10 | glpD | Reverse | ATCTTGCCCCCAAACACTGAA |
| 11 | Phd/yef | Forward | AGCAACATCATATTCGGCTTTCTTG |
| 12 | Phd/yef | Reverse | CGCTGGGCATCCTGTAGAAA |
| 13 | relB/dinJ | Forward | TGAAGATGCTTTTGCTTTTCTCAGT |
| 14 | relB/dinJ | Reverse | AATTGGGGATTACCCCTGCC |
| 15 | ftsI | Forward | ATCGTGCCTTATTTGCGGGT |
| 16 | ftsI | Reverse | CAAAAACTGGAGCGGCAACC |
| 17 | mrcA | Forward | TCGGTGAAAATGAAGGGCGT |
| 18 | mrcA | Reverse | GTTTTCACCACGCGACGAAC |
| 19 | mrdA | Forward | ACTTGCGCCGGATAAGTGTT |
| 20 | mrdA | Reverse | GAAAAAGCTGTGCAGGACGG |
| 21 | 16S | Forward | TGTGAAATCCCCGAGCTTAAC |
| 22 | 16S | Reverse | TATTAGGCCAGATGGCTGC |
| 23 | adeb | Forward | CCGCATCACCTTGAACATAAAC |
| 24 | adeB | Reverse | GGTGCTATGGGCGTTAGTATT |
| 25 | adeJ | Forward | CCATTGCTTTCATGGCATCACCAGA |
| 26 | adeJ | Reverse | AGCCGTATGATGCCTGAAGACTTA |
| 27 | adeF | Forward | CCGGTCGTTTAGAAGCAATG |
| 28 | adeF | Reverse | TTGCGGTATATGTTACCTGTGC |
| 29 | adeG | Forward | GGTCGGGCTAGCCTGTAAAA |
| 30 | adeG | Reverse | CTTGCTTCAACGGCTGCTTT |

**Methods:**

**Determination of antibiotic concentrations for persister isolation**

To determine the appropriate concentration of antibiotics for isolating persisters, the minimum inhibitory concentrations (MICs) of various antibiotics were first established for *A. baumannii* AYE and other strains used in this study. Following this, a range of antibiotic concentrations (10X, 20X, 25X, 50X, 75X, 100X, and 150X MIC) was tested to identify the minimum concentration at which a biphasic kill curve was observed over 24 hours of treatment.

Cultures were grown to an OD_600_ of 0.5 (corresponding to ~10⁷ CFU/mL). Log-phase cells were then treated with the respective antibiotic concentrations, and colony-forming units (CFU) were enumerated at specific time intervals for 24 hours. The biphasic kill curve was characterized by an initial rapid decline in CFU (representing the killing of actively growing cells), followed by a plateau phase indicating the survival of a subpopulation of dormant persister cells. The lowest antibiotic concentration resulting in a biphasic curve after 24 hours of treatment was selected for subsequent persister isolation experiments.

**Determination of cross-tolerance of persisters to other antibiotics**

To assess cross-tolerance, *A. baumannii* AYE persisters were isolated and exposed to rifampicin (40 µg/mL), levofloxacin (40 µg/mL), tigecycline (10 µg/mL), and polymyxin B (2.5 µg/mL) for 8 hours at 37 ºC. Log-phase cells were treated in parallel as controls. Following treatment, surviving cells were enumerated by spotting on LB agar plates.

The percentage survival of persisters and log phase cells upon treatment with these antibiotics was calculated by comparing the number of viable cells before and after antibiotic exposure.

**Assays under mechanism based screening to identify potential anti-persister compounds.**

**Outer Membrane Permeability Assay:** Outer membrane permeability was assessed using N-phenyl-1-naphthylamine (NPN). *A. baumannii* AYE were grown to 0.5 OD_600_ and washed thrice with 1X PBS and resuspended in 5 mM HEPES to 0.3 OD_600_. These cells at a volume of 200 µl were incubated with 0.25X MIC of the compounds for 30 minutes in half-area black plate (Corning) at 37 ºC. NPN (10 µM) was added to each well after incubation, and fluorescence was immediately measured at excitation/emission wavelengths of 350/420 nm using a SpectraMax M2e plate reader.

**Membrane Permeability Assay:** Membrane permeability was measured using SYTOX Orange^TM^ nucleic acid stain. *A. baumannii* AYE were grown to 0.5 OD_600_ and washed thrice resuspended in 1X PBS to an OD_600_ of 0.3. SYTOX Orange (1 µM) was added to the cells with constant stirring to stabilize the dye. 200 µl the dye-loaded cells and 0.25X MIC of the compounds were added to 96-well ~~opaque~~ half-area black plates and incubated for 30 minutes at 37 ^O^C. Fluorescence was measured after 30 minutes at excitation/emission wavelengths of 488/570 nm.

**ROS Generation Assay:** *A. baumannii* AYE were grown to 0.5 OD_600_ and washed thrice and resuspended in 1X PBS to an OD_600_ of 0.3, and incubated with Dichloro-dihydro-fluorescein diacetate (H_2_DCFHDA) at 10 µM for 30 minutes at 37ºC. Dye-treated cells were incubated with 0.25X MIC of the compounds in 96-well half-area black plates at 37ºC for 2 hours, and fluorescence was measured at excitation/emission wavelengths of 485/528 nm.

**Ethidium Bromide Accumulation Assay:** *A. baumannii* AYE cells were grown to 0.5 OD_600_ and washed thrice and resuspended in 1X PBS to an OD_600_ of 0.3, and incubated with ethidium bromide (10 µg/mL) at 37ºC for 20 minutes. Cells were then added to 96-well black plates containing 0.25X MIC of the compounds, and fluorescence was measured at excitation/emission wavelengths of 480/610 nm. The efflux inhibitory potential of thymol against meropenem persisters of *A. baumannii* was also measured using a similar protocol. Persisters were isolated as described previously in the material and methods section in the main text, after treatment with meropenem. Results expressed as fluorescence of surviving cells.

**Membrane Potential Assay:** *A. baumannii* AYE were grown to 0.5 OD_600_ and washed thrice and resuspended in 1X PBS to an OD_600_ of 0.3. Cells were incubated with DiBAC_4_(3) at 10 µM for 30 minutes at 37ºC. Cells were added to 96-well black plates containing 0.25X MIC of the compounds, and fluorescence was recorded at excitation/emission wavelengths of 490/516 nm.

For each experiment, the 200 µl of *A. baumannii* was added to 96-well plates and absorbance was measured at OD_600_. Relative fluorescence units (RFU) were calculated by dividing the fluorescence values obtained by the absorbance values of cells. Relative increase in fluorescence compared to untreated controls was plotted.

***In-vivo* murine infection**:

We used an acute skin wound infection model to determine the efficacy of thymol treatment in the eradication of meropenem persisters of *A. baumannii* using a protocol described earlier with minor modifications (1)(3). BALB/c mice were procured at the age of 5 weeks and housed for 7 days with free access to food and water.  Mice were provided with a 12 h day–night light cycle. Mice were immunocompromised on day −4 and day −1 by injecting cyclophosphamide (150 mg/kg body wt.; I.P.) (TCI Chemical, Japan). On day 0, mice were anesthetized with a 1:1 dose of ketamine (75 mg/kg body wt.; I.P.) (Themis Medicare, Ltd.) and xylazine (16 mg/kg body wt.; I.P.) (Indian Immunologicals, Ltd., India). While the mice were anesthetized, a 6 mm excision wound was created using a sterile biopsy punch (Mentok Healthcare, Pvt. Ltd.). To prevent skin contraction and maintain uniform wound size, a silicon splint (2mm thick) was attached around the entire wound area using medical-grade *n*-butyl cyanoacrylate glue (Eminent Chemtech, Pvt. Ltd., India). Then, meropenem persisters of *A. baumannii* AYE at a concentration of 10^6^ CFU/ml was seeded on the wound and allowed to fully dry before addition of 40μl of cell-free medium without or with antibiotics (with 50 μg/ml meropenem alone or in combination with thymol 128 µg/ml). After full drying, the wound site was bandaged firmly with medical gauze. After the mice were housed overnight, the scab on the wound was removed and homogenized. The lysates were serially diluted in sterile PBS and plated on Leeds Acinetobacter agar plates to enumerate surviving colonies.

**Supplementary References:**

1. Vineet D, Rinki G, Ranjana P. 2021. Targeting Superoxide Dismutase Confers Enhanced Reactive Oxygen Species-Mediated Eradication of Polymyxin B-dib Acinetobacter baumannii Persisters. Antimicrob Agents Chemother 65:10.1128/aac.02180-20.

2. Kumar A, Dalton C, Cortez-Cordova J, Schweizer HP. 2010. Mini-Tn7 vectors as genetic tools for single copy gene cloning in Acinetobacter baumannii. J Microbiol Methods 82:296–300.

3. Saini M, Gaurav A, Kothari A, Omar BJ, Gupta V, Bhattacharjee A, Pathania R. 2023. Small Molecule IITR00693 (2-Aminoperimidine) Synergizes Polymyxin B Activity against Staphylococcus aureus and Pseudomonas aeruginosa. ACS Infect Dis 9:692–705.
